# Advanced Sperm Selection Techniques for Assisted Reproduction

**DOI:** 10.3390/jpm14070726

**Published:** 2024-07-04

**Authors:** Federica Cariati, Maria Grazia Orsi, Francesca Bagnulo, Daniela Del Mondo, Luigi Vigilante, Martina De Rosa, Romualdo Sciorio, Alessandro Conforti, Steven Fleming, Carlo Alviggi

**Affiliations:** 1 Department of Public Health, School of Medicine, University of Naples Federico II, 80131 Naples, Italy; federica.cariati@unina.it (F.C.); lui.vigilante@gmail.com (L.V.); martina-derosa@libero.it (M.D.R.); alviggi@unina.it (C.A.); 2 Fertility Unit, Maternal-Child Department, AOU Federico II Polyclinic, 80131 Naples, Italy; francesca.bagnulo@unina.it; 3 Department of Neuroscience, Reproductive Science and Odontostomatology, University of Naples Federico II, 80131 Naples, Italy; orsimariagrazia.98@gmail.com (M.G.O.); alessandro.conforti@unina.it (A.C.); 4 Department of Molecular Medicine and Medical Biotechnology, University of Naples Federico II, 80131 Naples, Italy; danieladelmondo@gmail.com; 5 Fertility Medicine and Gynaecological Endocrinology Unit, Department Woman Mother Child, Lausanne University Hospital, 1011 Lausanne, Switzerland; 6 Discipline of Anatomy & Histology, School of Medical Sciences, University of Sydney, Sydney, NSW 2050, Australia; steven.fleming@sydney.edu.au

**Keywords:** male infertility, sperm selection, assisted reproductive technology, oxidative stress

## Abstract

Male infertility accounts for approximately 40% of infertility cases. There are many causes of male infertility, including environmental factors, age, lifestyle, infections, varicocele, and cancerous pathologies. Severe oligozoospermia, cryptozoospermia, and azoospermia (obstructive and non-obstructive) are identified as severe male factor infertility, once considered conditions of sterility. Today, in vitro fertilization (IVF) techniques are the only treatment strategy in cases of male factor infertility for which new methodologies have been developed in the manipulation of spermatozoa to achieve fertilization and increase success rates. This review is an update of in vitro manipulation techniques, in particular sperm selection, emphasizing clinical case-specific methodology. The success of an IVF process is related to infertility diagnosis, appropriate choice of treatment, and effective sperm preparation and selection. In fact, selecting the best spermatozoa to guarantee an optimal paternal heritage means increasing the blastulation, implantation, ongoing pregnancy and live birth rates, resulting in the greater success of IVF techniques.

## 1. Introduction

Infertility is a widespread problem that affects approximately one in six couples of childbearing age. The World Health Organization (WHO) identifies infertility as failure of a couple to conceive naturally after 12–24 months of unprotected sexual intercourse [1,2]. Approximately 85% of infertile couples have an identifiable cause, the most common etiologies including ovulatory dysfunction, male infertility and tubal disease; the remaining 15% of couples suffer from idiopathic infertility, without a determinable organic cause. Lifestyle and environmental factors, such as smoking, environmental toxins and obesity, can negatively affect fertility, but infertility can also be an indicator of an underlying chronic disease [3]. Recently, some studies have shown that the rate of male infertility is continually growing, in step with the increase in cases of testicular tumors [4]. Male infertility represents an enormously widespread phenomenon in the population and has highly heterogeneous causes, which may be related to pre-testicular (alterations of the hypothalamic–pituitary axis), testicular and post-testicular conditions (urogenital obstructions, vasectomy and dysfunction of accessory glands). Disorders of male physiology, such as low testosterone concentrations or low sperm count, occur in 30% of infertile couples [5]. A couple may also have multiple factors contributing to infertility; therefore, an evaluation for male factor infertility should be performed at the same time as the female evaluation [6,7]. Additionally, as new scientific perspectives develop, some studies have shown a greater bacterial presence in infertile subjects demonstrating how the seminal microbiota can also be studied for the diagnosis of male infertility, hypothesizing that its management could provide a potential solution to the cause of infertility [8,9]. Assisted reproductive technology (ART) has improved and in vitro fertilization (IVF) and its variants are increasingly used to treat almost all causes of male infertility [10,11]. Advances in ART are of two types: the incremental optimization of existing techniques and the development of new technologies. IVF techniques are currently a treatment strategy in cases of female factor infertility, and in cases of male factor and idiopathic infertility. Intrauterine insemination (IUI), and embryo transfer following IVF and intracytoplasmic sperm injection (ICSI) are three treatment techniques (first–second–third level) based on the type and cause of infertility. The treatment approach for each couple is personalized according to the type of infertility encountered [12]. Although there is no formally recognized definition for mild male factor infertility, IUI is a first-line treatment strategy and, in 2016, Cissen and collaborators [13] analyzed 10 randomized clinical trials (RCTs) involving 757 cases of male infertility without finding substantial evidence of a significant difference between IUI and planned sexual intercourse. In cases of substantial or severe male factor infertility (SMF), a typical cycle of ART includes gonadotropin stimulation in the woman, followed by insemination of oocytes aspirated from multiple ovarian follicles, with the aim of selecting spermatozoa to ensure fertilization with IVF or ICSI [2]. Severe oligozoospermia (<5 × 10^6^ spermatozoa/mL of ejaculate), cryptozoospermia (spermatozoa only observable after centrifugation and microscopic observation of the sperm pellet), obstructive and non-obstructive azoospermia (1% of the general male population and 10–15% of the infertile male population) are identified as forms of SMF [14]. In the past, patients with SMF were considered sterile; today couples affected by this form of infertility resort to ICSI [15], preceded in the case of obstructive or non-obstructive azoospermia by microsurgical epididymal sperm aspiration (MESA), percutaneous epididymal sperm aspiration (PESA), testicular sperm aspiration (TESA) or testicular sperm extraction (TESE) [16]. Raman spectroscopy represents an innovative and valuable tool to assist surgeons during micro-TESE, helping to improve sperm retrieval. It is able to non-invasively differentiate seminiferous tubules with complete and incomplete spermatogenesis [17]. Testicular tissue is subjected to mechanical dissociation or enzymatic digestion and sperm are isolated under light microscopy for immediate use in an ICSI technique or for cryopreservation. Moreover, promising studies have been published on sperm selection and recovery after TESE using fluorescence-activated cell sorting (FACS). The aim is to increase the efficiency of the technique in terms of quantity of recovered spermatozoa, especially in non-obstructive azoospermia [18]. FACS is potentially useful for selecting spermatozoa with good DNA integrity [19]. Strassburger and colleagues showed a reduction in the success rates of ICSI in SMF patients [20]; subsequently, further studies carried out on a cohort of 1219 consecutive ART cycles highlighted that SMF infertility does not influence the quality of the blastocyst obtained by ICSI [21], leading to reflection on the genetic quality of the spermatozoa. However, the relative contribution of severe sperm factors and ICSI, commonly used to overcome reproductive difficulties in affected men, is not really known. Recent studies have demonstrated that ICSI followed by assisted oocyte activation (ICSI-AOA) is advantageous in couples with severe teratozoospermia, including globozoospermia, and in cases of repeated failures post-ICSI [22,23,24]. Therefore, IVF/ICSI can cure most cases of moderate to SMF infertility that cannot be resolved with medical or surgical approaches. However, the success of ART is not related to the diagnosis and insemination technique alone, but also to sperm preparation and selection [25]. Natural sperm selection in humans is a rigorous process; the human ejaculate is made up of a heterogeneous pool of spermatozoa and natural selection translates into only about a thousand of the 10^7^ ejaculated spermatozoa being capable of reaching and fertilizing the oocyte [26]. With the introduction of IVF, the need arose to develop a wide range of sperm selection techniques [25], in order to mimic in vitro the selection that the male gamete undergoes during transit through the female reproductive tract [27]. As an alternative to the conventional swim-up (SU) and discontinuous density gradient centrifugation (DGC) methods, advanced sperm selection techniques have been developed capable of selecting sperm with reduced apoptosis and increased sperm DNA integrity, by virtue of their ability to bind molecules present on the sperm or oocyte surface, respectively, such as phosphatidylserine or hyaluronic acid (HA). However, to date, no clear results translating into higher success rates of ART have been achieved [28]. The need to select the best spermatozoa in order to guarantee an optimal paternal heritage via ART is closely related to the need to protect and guarantee the health prospects for both the woman during gestation and to the unborn child in the short and long term [29,30]. The development of new techniques capable of selecting a good population of spermatozoa is essential for artificial insemination, IUI, IVF, ICSI, and sperm cryopreservation to increase success rates in ART cycles [28,29]. The objective is discarding low-quality spermatozoa, in terms of motility and morphology, but also reducing the sperm DNA fragmentation that is associated with a high rate of spontaneous abortion [31]. This review analyzes conventional and advanced sperm selection techniques for ART, clarifying the principles they are based upon. A comparison between the advantages and disadvantages of each standard or experimental method could lead to new questions and further studies.

## 2. Sperm Manipulation Techniques in IVF 

### 2.1. Swim-Up and Density Gradient Centrifugation

The SU technique is performed in two ways: SU from a physical layer and SU from a sperm pellet [25]. Both are based on the ability of spermatozoa with good motility to migrate spontaneously in a suitable culture medium, usually placed over a portion of the semen sample, during an incubation period at a 45° angle; but in the case of pellet SU, over a sperm pellet obtained after centrifugation of the semen sample [32]. It is advisable to use the pellet SU in cases of oligoasthenoteratozoospermia since, compared to layer SU, it enables a more concentrated final suspension of spermatozoa to be obtained [27]. SU is recommended both for IVF and ICSI in cases of asthenozoospermia [1]. The DGC method separates spermatozoa according to their density, size and shape, and allows the selection of motile spermatozoa with good morphology, which will be able to reach the bottom of the centrifuge tube during the centrifugation process, progressing through colloidal silica solutions of increasing density [25,33]. In contrast, the immobile, morphologically abnormal spermatozoa, non-gametic cells and debris will stop between the discontinuous gradients created with different density layers of colloidal silica. The compounds used to construct the gradients must not be toxic, must be stable in solution and must not alter the biological material [34]. Ficoll and Percoll (mixed compounds, such as colloidal silica coated with polyvinylpyrrolidone) were among the most-used gradient materials in the past [35]. Both SU and DGC can remove spermatozoa with double-strand DNA damage and highly damaged DNA, but the two-tailed single gel electrophoresis comet assay and the sperm chromatin dispersion test have shown that SU is less efficient than DGC in selecting sperm devoid of single-stranded DNA damage [36]. In 2021, the SU and DGC techniques were compared in normozoospermic semen samples, and high levels of oxidative stress, increased hyperactivation, and tyrosine phosphorylation were observed in the samples treated with DGC; additionally, sperm separated by DGC were found to be more capacitated than SU sperm, so DGC should be preferred for ICSI, while SU should be preferred for techniques such as IUI and IVF (Table 1) [37]. Raad and co-workers demonstrated that SU-obtained spermatozoa possessed fewer vacuoles in their heads compared to DGC [38]. In addition, with DGC a higher sperm recovery rate is achieved, whereas a higher rate of progressive motility is achieved following SU [39]. Comparing SU and DGC, no significant differences in fertilization, good-quality embryo, or blastocyst formation rates were found in IVF/ICSI cycles [40]. However, the conventional selection methods mentioned above are not entirely effective in cases of severe oligoasthenozoospermia, where a modified selection method consisting of a reduced volume of gradient (mini gradient) appears to be optimal [41]. SU and DGC allow us to obtain a sperm population with a low percentage of apoptotic spermatozoa and, furthermore, a recent meta-analysis showed that there is no evidence to favor one methodology over the other [41,42]. In recent years, therefore, the need has arisen to develop new selection methodologies, paying particular attention to the integrity of the sperm DNA, avoiding the generation of reactive oxygen species (ROS), supporting the maturation of the sperm plasmalemma and preventing apoptosis, to improve the outcome of ART. Among advanced methods, the following means of sperm selection have been exploited: the electrical zeta potential, magnetic-activated cell sorting (MACS), the hemi-zona assay (HZA), selection based on the binding of spermatozoa to HA and microfluidics (shown in Table 2).

### 2.2. Zeta Potential Method

**Figure 1:**
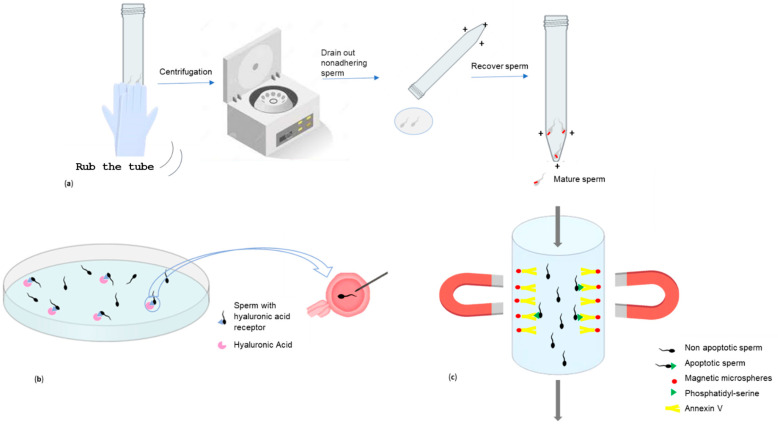
Schematic representation of sperm selection techniques. Representation of the zeta potential method (**a**); graphic representation of the selection technique with hyaluronic acid (**b**); magnetic-activated cell sorting (MACS) representation (**c**).

**Figure 2:**
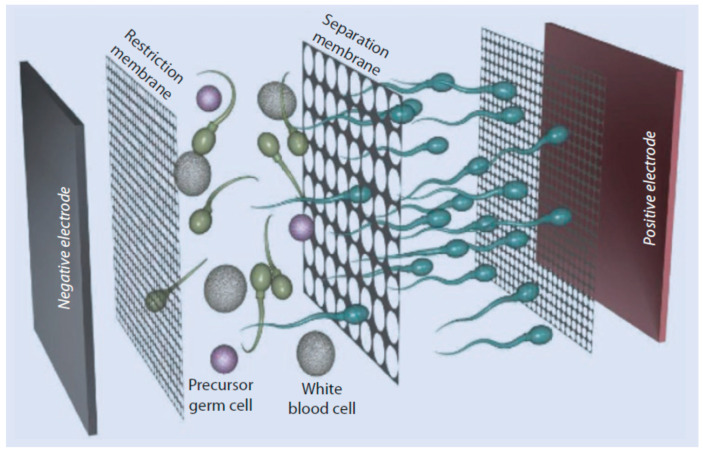
Diagram illustrating a schematic representation of sperm electrophoretic mobility.

**Figure 3:**
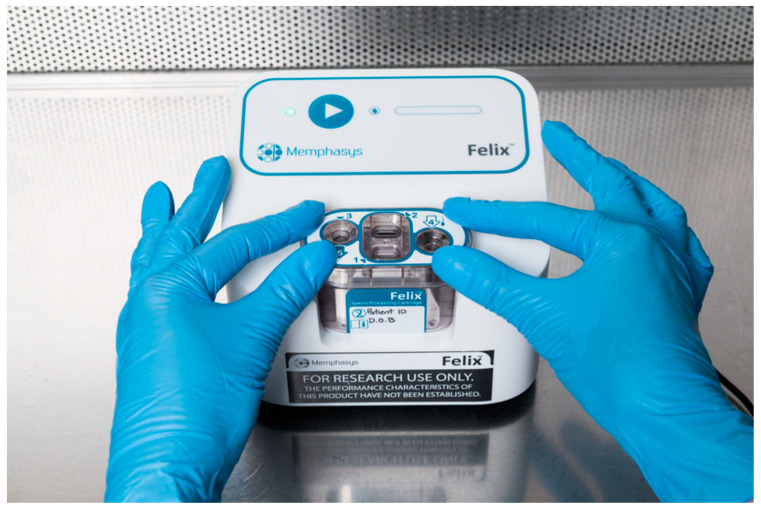
Felix system. Electrophoretic sperm isolation device.

The zeta potential method (Figure 1a) exploits the characteristic negative electrical charge of mature spermatozoa, which is between −16 and −20 mV [55]. It is believed that this negative charge is due to the presence of the glycosylphosphatidylinositol-anchored sialoglycoprotein, CD52, which is acquired during epididymal transit and located within the glycocalyx on the sperm plasmalemma [56,57,58]. Essentially, the zeta potential technique involves the electrostatic adhesion of sperm to a positively pre-charged plastic centrifuge tube. It is a simple technique to perform, inexpensive and enables the rapid recovery of mature spermatozoa with intact DNA and normal morphology: those spermatozoa capable of binding to the walls of a centrifuge tube, previously positively charged. A disadvantage of the zeta method is the recovery of a low number of spermatozoa, making it unusable in cases of oligozoospermia [59]. It has been observed, although it needs to be confirmed with further studies, that by combining the zeta potential technique with DGC, pregnancy rates following ICSI are better than individual procedures, and the zeta method was found to be more efficient in the selection of spermatozoa free of DNA fragmentation [43]. In 2005, Ainsworth and collaborators developed a novel electrophoretic system for the isolation of human spermatozoa that consists of a chamber containing positive and negative poles between which a polycarbonate filter with 5 μm pores is placed (Figure 2); when current is applied, only negatively charged motile, viable, morphologically normal spermatozoa with low levels of DNA damage will pass through the filter [60]. In 2008, Fleming and co-workers demonstrated that there were no differences between DGC and the electrophoretic system in the preparation of sperm for IVF, although the electrophoretic system was much quicker [61]. A commercial electrophoretic sperm isolation device has since been developed: the Felix™ system (Memphasys reproductive biotechnology, Homebush West, Australia), a second generation of the prototype known as the Cell Sorter 10, or CS10 (Figure 3), is a successful device for preparing spermatozoa, offering both speed and spermatozoa with improved progressive motility and reduced DNA damage. However, one drawback is its lower recovery rate (18.7%), compared to DGC, potentially making it less suitable for severe oligozoospermic samples [32]. Furthermore, both the DGC and Felix methods isolate vital and highly motile sperm, although the Felix method retrieves a lower number of sperm but with less DNA damage, and does so in just six minutes (the DGC method takes 40 min) [62].

### 2.3. Hyaluronic Acid Binding

Another established approach for sperm selection exploits the ability of mature spermatozoa to bind HA, a molecule normally present within the extracellular matrix of the cells of the cumulus oophorus, thus representing a barrier that can only be overcome by spermatozoa that have receptors capable of binding to it (Figure 1b) [63]. Mature spermatozoa selected with HA are viable, free of DNA fragmentation, non-apoptotic, and with a frequency of chromosomal diploidies within normal limits [64]. To date, the physiologic ICSI (PICSI) dish^®^ and SpermSlow™ (CooperSurgical Fertility Solutions, Ballerup, Denmark) are the two commercially available sperm selection systems relying upon binding to HA; Parmegiani and co-authors found no statistically significant difference regarding the percentage of good quality embryos obtained between the two of them [65]. With the PICSI dish, in particular, only those spermatozoa with motile tails that appear adhered by their heads to the bottom of the Petri dish pre-treated with HA hydrogel would be selected and injected [66]. Interestingly, the HABSelect trial conducted in the UK, a multicenter RCT of 2772 non-selected couples with a variety of male factor etiologies, demonstrated that sperm selection with the PICSI dish significantly reduced the miscarriage rate compared to standard ICSI (4.3% vs. 7.0%, *p* = 0.003), with a trend also towards a higher live birth rate (27.4% vs. 25.2%, *p* = 0.18) [44]. 

### 2.4. Hemi-Zona Assay (HZA)

In order to fertilize the oocyte, the ejaculated spermatozoa must bind the zona pellucida and therefore the HZA was developed, aimed at selecting spermatozoa capable of adequately carrying out this binding so as to evaluate their fertilizing potential [67,68]. It is a test that requires two halves of a zona pellucida from a non-fertilized human oocyte: one will be used for a positive control using seminal fluid from fertile men, while the other will be used to test the patient’s semen [67,69]. The hemi-zona index (HZI: number of ligated spermatozoa of the patient/number of ligated spermatozoa of the fertile man × 100) must be above 30% for IUI likely to be successful [45]. This test has been shown to be useful in identifying patients who may experience difficulty achieving fertilization through IVF treatment [70].

### 2.5. Magnetic-Activated Cell Sorting (MACS)

MACS (Figure 1c) is another innovative method, which exploits the characteristics of the sperm plasmalemma. This technique allows the removal of apoptotic spermatozoa from the seminal fluid, with the aid of a column subjected to a magnetic field, inside which there are paramagnetic micro-beads conjugated to annexin V, a protein binding various phospholipids, including phosphatidylserine [71]. The externalization of phosphatidylserine, from the internal to the external side of the sperm membrane, is associated with an early apoptotic process. Therefore, annexin V is an excellent biomarker of apoptotic spermatozoa, which will be retained along the walls of the column, not being selected for downstream applications [72]. In contrast, non-apoptotic spermatozoa will pass through the column and can be used for ART, optimizing results [73]. Studies have shown that MACS improves sperm motility and cryo-survival rates after sperm cryopreservation [74]. Furthermore, MACS is more efficient than PICSI in the elimination of spermatozoa with fragmented DNA in cases of idiopathic infertility and it significantly increases pregnancy and live birth rates in patients with high sperm DNA fragmentation compared to the control group (60.7% vs. 51.5% and 47.4% vs. 31.2%, respectively) [46,47]. In addition, DGC followed by MACS (DGC-MACS) dramatically reduces the rate of spontaneous abortion in patients with a sperm DNA fragmentation level ≥ 30% in ICSI cycles [75]. Furthermore, DGC-MACS may increase implantation rates compared to DGC alone in teratozoospermia, although further studies are needed [76]. In 2023, Bibi and colleagues demonstrated that DGC-MACS, compared to DGC alone, SU, and DGC followed by SU, provides a higher percentage of viable spermatozoa with normal morphology and intact DNA in teratozoospermic patients, increasing the success rates of ART [48].

### 2.6. Hypo-Osmotic Swelling Test (HOST)

The HOST was proposed as a method which allows the selection, starting from a basal seminal sample with severe asthenozoospermia, of viable spermatozoa with intact cell membranes: those which in hypo-osmotic solution show a swelling of the cytoplasm (due to solvent ingress) and an apparent curling of the tail when observed under the microscope [69,77]. The functional integrity of the sperm membrane is essential for spermatozoa to be able to withstand the osmotic challenges from the hyperosmotic environment of the cauda epididymis to the almost iso-osmotic female genital tract [78,79].

This test could, therefore, increase fertilization rates in ICSI cycles in which motile spermatozoa are not recovered in the ejaculate or following surgical sperm recovery (SSR); it represents a valid alternative to the vital staining technique, which should be avoided when spermatozoa must be selected for ICSI [1,80]. However, the characteristic swollen tail of the spermatozoon could make its aspiration into the microinjection pipette difficult [50]. HOST could be useful in cases of non-motile spermatozoa as, for example, in Kartagener’s syndrome or after SSR [81]. 

### 2.7. Laser-Assisted Immobile Sperm Selection (LAISS)

A tail reaction similar in appearance to that observed in the HOST is achieved in laser-assisted immobile sperm selection (LAISS), where a laser shot is fired at the sperm tail, causing it to curl only in viable spermatozoa. In this way, therefore, it is possible to select viable spermatozoa in cases of complete asthenozoospermia [82]. In asthenozoospermic samples, the use of LAISS results in significantly higher fertilization and embryo cleavage rates compared to those obtained from randomly selected spermatozoa [50]. Today, there are more and more technologies that exploit the laser system both in the selection of viable spermatozoa and in immobilization before ICSI, thus eliminating the use of chemical substances that might be harmful to them. However, their high cost and complexity are the main disadvantages that will need to be addressed [83].

### 2.8. Microfluidics

Microfluidics deals with the flow of very small quantities of liquid, using micrometric capillaries and represents a new sperm selection method. Since microfluidics can imitate what happens in the female reproductive tract, it allows the separation of motile spermatozoa from non-motile ones and from other cells [34,84]. Over time, numerous microfluidic devices have been developed, from simple single-channel ones to “k”-type ones with multiple apertures [85]. For example, a new microfluidic device, FertDish, enables the selection of high-quality spermatozoa with low DNA fragmentation using microfluidics in a dish for ICSI [86]. Indeed, compared to conventional techniques currently used in IVF laboratories, microfluidics more efficiently select motile spermatozoa with intact DNA [28]. This has also been demonstrated using new microfluidic devices based on chemotaxis (created using cumulus cells) and thermotaxis [87]. The microfluidic sperm sorter (MSS) chip is an innovative microfluidic platform that enables the selection of sperm with high DNA integrity and fewer ROS [88]. This device consists of two chambers separated by a polycarbonate filter with various diameters. Specifically, only sperm with good motility and morphology are able to swim to the upper chamber. The MSS provides a higher number of higher quality blastocysts compared to DGC [51]. Vasilescu and co-workers developed a 3D-printed spiral microchannel device as a viable alternative for sperm isolation from mixed cell suspensions, allowing high sperm recovery [89]. Mixed cell suspensions are injected into the device using syringes, and the spermatozoa that have successfully migrated inside the spiral channel are subsequently recovered from the outlet channels. The application of this approach could be introduced in SSR procedures. Furthermore, comparing SU with a microfluidic system, the latter selects spermatozoa with significantly lower DNA damage [90]. For example another microfluidics-based device is the FERTILE^®^-Chip, which allows for the selection of sperm with a significantly lower percentage of double-stranded DNA fragmentation compared to fresh ejaculate or SU [91]. Moreover, this device can improve the formation and euploidy rates of blastocysts after ICSI compared to the DGC method [92]. Aydin and co-workers demonstrated that the Fertile Chip, in patients with male infertility undergoing ICSI, also yields significantly higher implantation and live birth rates compared to the SU procedure [93]. Mirsanei and collaborators in 2022, demonstrated that microfluidic chips, in couples with fertilization failure following ICSI, enabled sperm selection with improved motility and morphology, and with a lower DNA fragmentation rate compared to the DGC method; therefore, improving the fertilization rate [94].

### 2.9. Motile Sperm Organelle Morphology Examination (MSOME)

MSOME was introduced in 2002 by Bartoov and colleagues and is an innovative technique that allows for a more detailed analysis of sperm morphology by virtue of a magnification of up to 6300x, obtained by combining Nomarski optics with digital magnification [95]. Sperm are evaluated based on the morphology of the nucleus, neck, acrosome, post-acrosomal zone, mitochondria and tail [96]. It has been observed that the presence of large nuclear vacuoles are indicative of spermatozoa with anomalous chromatin packaging, an indication that can be highlighted by MSOME, thus allowing the elimination of such spermatozoa [97]. The application of MSOME to ICSI has given rise to intracytoplasmic morphologically selected sperm injection (IMSI) which allows sperm with the best morphology to be used in microinjection techniques [98]. Although, in general, there are no significant differences in the degree of fertilization after ICSI and IMSI, the latter has proven effective in cases of repeated failures in ICSI cycles [52,53]. Unfortunately, IMSI appears to be disadvantageous overall due to being a longer procedure [52].

### 2.10. Additional Experimental Methods: Sperm Tail Flexibility Test (STFT), Birefringence and Artificial Intelligence

The sperm tail flexibility test (STFT) is a test based on the flexible tail characteristic of viable, though immotile, spermatozoa, and allows their preferential selection [50]. In particular, the tail is defined as flexible when it moves independently from the movement of the head when manipulated with an ICSI pipette. The STFT can be an alternative test to the HOST [99]. Birefringence is a new technique that allows the selection of spermatozoa with a compact nucleus and normal acrosome using polarization microscopy [99]. A study by Magli and co-workers observed that immotile birefringent sperm in asthenozoospermic patients had a higher fertilization rate compared to non-birefringent ones in ICSI [54]. Along that line, a novel approach, a joint probabilistic data association filter (JPDAF) has been developed, which is a fully automated multi-sperm tracking algorithm capable of simultaneously detecting and tracking hundreds of sperm within video frames, accurately measuring motility over time. Unlike the CASA system, it has the capability to monitor sperm swimming behavior in close proximity and during apparent cell-to-cell collisions [100,101].

## 3. Sperm Selection Techniques and Reactive Oxygen Species

ROS play a significant physiological role as they are involved in capacitation, hyperactivation, and the acrosome reaction [102]. The presence of antioxidant molecules in seminal fluid, sperm, and the male reproductive tract maintain ROS in a balanced state. However, there can be situations such as genetic factors, nutritional imbalances, and pathological conditions that may lead to an antioxidant deficiency, causing reduced fertility [103]. Sperm DNA damage mediated by ROS is involved in 30–80% of male infertility cases [104]. An increase in ROS can lead to oxidative stress, compromising the integrity of the sperm membrane, causing DNA fragmentation and reduced sperm motility; this excess of ROS can be caused by exogenous factors such as centrifugation, commonly used in conventional sperm selection methods like SU and DGC [105]. Elevated levels of ROS-positive cells were observed in centrifugation-dependent techniques, particularly in DGC and DGC/SU, in comparison to raw semen [38]. The absence of centrifugation in microfluidic devices minimizes sperm exposure to ROS, allowing for the retrieval of sperm with high DNA integrity and significantly lower levels of ROS production compared to SU [88]. The electrophoretic sperm separation device, Felix, also does not involve centrifugation and allows for the selection of spermatozoa with lower DNA oxidative damage compared to sperm prepared with DGC [32]. Talevi and co-workers demonstrated that normozoospermic and oligozoospermic samples treated in vitro with antioxidants such as zinc, D-aspartate, and coenzyme Q10 maintain their motility, exhibit reduced DNA fragmentation, and show low lipid peroxidation compared to untreated samples [106]. Recent studies have highlighted that excessive accumulation of reducing agents also leads to increased levels of ROS (reductive stress), causing damage to spermatozoa [107]. Indeed, the use of antioxidants to address male infertility caused by oxidative stress has been considered a primary strategy [108].

## 4. Conclusions

SU and DGC are the most commonly used sperm preparation methods, as they are fast, easy and economical, therefore satisfying many of the requirements required for preparation [109]. Although there are new selection methods to date, none of them appear to be a “gold standard” technique, but experimental evidence has shown that improvements have been made in the field of ART, compared to the conventional methods of SU and DGC [34,110]. The results obtained certainly represent the foundation on which to base future innovations to offer the maximum possibility of conception to infertile couples. Methodologies that mimic the selection process in the female reproductive tract and do not solely rely on characteristics such as motility and density could result in the selection of spermatozoa with enhanced fertilization potential [111]. The embryologist’s challenge must be to choose, among many techniques, the one most suitable for the patient, without carrying out standard protocols regardless of patient etiology. The benefit of a personalized approach lies precisely in being able to obtain high fertilization rates, one of the objectives of IVF. Certainly, a crucial future perspective involves continuous research into the molecular and biological mechanisms underlying male infertility, in order to provide new insights for future treatments [112]. Another future perspective is the research for tests capable of diagnosing the cause of male infertility (such as genetic mutations, epigenetic factors, and oxidative stress), which is useful for the pursuit of appropriate treatment [113]. In 2023, for example, Zhang and co-authors identified DNA methylation markers associated with bull fertility through sequencing [114]. Furthermore, the implementation of nanotechnology-based assays and the discovery, in 2022, that normozoospermic human spermatozoa behave as a viscoelastic fluid rather than a purely viscous fluid, can offer advantages in the diagnosis and treatment of infertility [115,116]. Recent studies have demonstrated the correlation between sperm DNA fragmentation and poor embryonic development, higher miscarriage and lower implantation rates, following IVF or ICSI [117,118]. Cariati and co-workers have also demonstrated the correlation between short sperm telomeres and chromosomal anomalies [119]. Clinical data from experimental selection methods are uncertain and consequently it remains difficult to choose an alternative to standard techniques such as SU or DGC. For this reason, further high-quality studies, considering several factors in addition to standard semen parameters as reported above, that are fast, inexpensive, easy, and without requiring multiple centrifugations should be tested in order to include them in clinical practice. The implications of findings regarding sperm selection techniques based on sperm quality could drive IVF procedures to increase success rates in terms of live birth or clinical pregnancy.

## Figures and Tables

**Table 1:** Different selection methods according to the sperm parameters.

Semen Alterations	Suggested Protocols
*Teratozoospermia, asthenozoospermia and oligozoospermia*	Swim up
*Teratozoospermia, asthenozoospermia and oligozoospermia*	Density gradient centrifugation
*Teratozoospermia*	Zeta potential
*Teratozoospermia*	Hyaluronic acid binding
*Teratozoospermia*	Hemi-zona assay
*Teratozoospermia*	Magnetic-activated cell sorting
*Severe asthenozoospermia*	Hypo-osmotic swelling test
*Complete asthenozoospermia*	Laser-assisted immobile sperm selection
*Teratozoospermia and asthenozoospermia*	Microfluidics
*Teratozoospermia*	Motile sperm organelle morphology examination
*Asthenozoospermia*	Birefringance
*Asthenozoospermia*	Sperm tail flexibility test
*Non-obstructive azoospermia*	Fluorescence-activated cell sorting

**Table 2:** Advantages and disadvantages of the use of individual sperm selection techniques.

Protocol	Advantages	Disadvantages	Description	Clinical Outcomes
Swim Up (SU)	Easy, economical recovery of highly motile spermatozoa	Not effective in cases of severe oligoasthenozoospermia	Motile spermatozoa will migrate in a suitable culture medium, placed over a portion of the semen sample	Comparing SU and DGC, no significant differences in fertilization, good-quality embryo, and blastocyst formation rates have been found in IVF/ICSI cycles [40]
Density Gradient Centrifugation (DGC)	Easy, economical recovery of highly motile spermatozoa with normal morphology	Not as effective in cases of severe oligoasthenozoospermia	Spermatozoa with a good morphology will reach the bottom of the centrifuge tube during centrifugation process, progressing through solutions of increasing density	
Zeta Potential	Selection of mature spermatozoa with intact DNA and normal morphology	Low efficiency	Spermatozoa with negative charge will bind to the walls of a centrifuge tube, previously positively charged	Combining the zeta potential technique with DGC, pregnancy rates following ICSI are better than individual procedures [43]
Hyaluronic Acid Binding (Ha)	Selection of viable non-apoptotic spermatozoa, free of DNA fragmentation, and with a frequency of chromosomal diploidy within normal limits	Low selection of motile spermatozoa with normal morphology at high magnification	Spermatozoa, with HA receptors will adhere with their heads to the bottom of Petri dishes pre-treated with HA hydrogel	Sperm selection with the PICSI dish significantly reduced the miscarriage rate compared to standard ICSI [44]
Hemi-Zona Assay (HZA)	Identification of spermatozoa capable of binding to the zona pellucida	Difficult to obtain donated human oocytes	Spermatozoa able to bind to glycoprotein receptors ZP3/ZP4 on the zona surface from a non-fertilized human oocyte can be counted as normal	The hemi-zona index must be above 30% for IUI likely to be successful [45]
Magnetic-Activated Cell Sorting (MACS)	Selection of non-apoptotic spermatozoa	Expensive	Non-apoptotic spermatozoa will pass through a column of annexin-V coated paramagnetic beads subjected to a magnetic field	MACS is more efficient than PICSI in the elimination of spermatozoa with fragmented DNA in cases of idiopathic infertility [46,47]. DGC-MACS, compared to DGC alone, SU, and DGC followed by SU, provides a higher percentage of viable spermatozoa with normal morphology and intact DNA in teratozoospermic patients [48]
Hypo-Osmotic Swelling Test (HOST)	Economical, easy, selection of viable spermatozoa with an intact plasmalemma	Difficult aspiration into an ICSI micropipette	Viable spermatozoa in hyposmotic solution will show a swelling of the cytoplasm due to osmosis	In cases of immotile spermatozoa, HOST and the sperm tail flexibility test enable selection of viable spermatozoa; however they work better in fresh than in cryopreserved spermatozoa [49]
Sperm Tail Flexibility Test (STFT)	Selection of viable but immotile spermatozoa	Not a standardized technique. Further studies are required	Spermatozoa are shaken with an ICSI needle	
Laser-Assisted Immobile Sperm Selection (LAISS)	No exposure of the spermatozoa to potentially harmful chemical substances. Selection of live spermatozoa in severe asthenozoospermia	Expensive, difficult	A laser shot is fired at the sperm tail to observe tail curling as an indication of viability	In asthenozoospermic samples, the use of laser results in significantly higher fertilization and embryo cleavage rates compared to those obtained from randomly selected spermatozoa [50]
Microfluidics	Recovery of motile spermatozoa with normal morphology and intact DNA	Expensive, not a standardized technique	Using micrometric capillaries, motile spermatozoa will separate from non-motile ones, and from other cells	Microfluidics provides a higher number of higher quality blastocysts compared to DGC [51]
Motile Sperm Organelle Morphology Examination (MSOME)	More detailed analysis of sperm morphology, high pregnancy rates	Long procedure, expensive	Spermatozoa are observed at a magnification of up to 6300x	There are no significant differences in the degree of fertilization after ICSI and IMSI [52,53]
Birefringence	Selection of spermatozoa with a compact nucleus and normal acrosome	Not a standardized technique. Further studies are required	Spermatozoa are observed under polarization microscopy	Immotile birefringent sperm in asthenozoospermic patients have a higher fertilization rate compared to non-birefringent ones in ICSI [54]
Fluorescence-Activated Cell Sorting (FACS)	Recovery of spermatozoa from a mixed sample (especially in non-obstructive azoospermia)	Expensive technique, high percentage of cell loss, time-consuming	Spermatozoa from seminal fluid labeled with fluorophore-conjugated antibodies fluoresce when excited by a laser and can be recovered	The alteration in cell viability due to fluorophores and antibodies limits the use of FACS in routine clinical practice [19]

## Data Availability

No data is available.
